# Author Correction: Structural basis for delta cell paracrine regulation in pancreatic islets

**DOI:** 10.1038/s41467-019-12258-7

**Published:** 2019-09-11

**Authors:** Rafael Arrojo e Drigo, Stefan Jacob, Concha F. García-Prieto, Xiaofeng Zheng, Masahiro Fukuda, Hoa Tran Thi Nhu, Olga Stelmashenko, Flavia Letícia Martins Peçanha, Rayner Rodriguez-Diaz, Eric Bushong, Thomas Deerinck, Sebastien Phan, Yusuf Ali, Ingo Leibiger, Minni Chua, Thomas Boudier, Sang-Ho Song, Martin Graf, George J. Augustine, Mark H. Ellisman, Per-Olof Berggren

**Affiliations:** 1 Lee Kong Chian School of Medicine, Nanyang Technological University, Singapore, 636921 Singapore; 2 Karolinska Intitutet, The Rolf Luft Research Center for Diabetes and Endocrinology, Stockholm, 171 77 Sweden; 3 Molecular Neurophysiology Laboratory, Signature Program in Neuroscience and Behavioral Disorders, Duke-NUS Medical School, Singapore, 169857 Singapore; 4 Bioinformatics Institute (ASTAR) and Image and Pervasive Access Lab (IPAL), Singapore, 138632 Singapore; 5 Sorbonne University, UPMC University, Paris, 75005 France; 6 Universidade Federal do Rio de Janeiro (UFRJ), Rio de Janeiro, 21941-901 Brazil; 7 Miller School of Medicine, University of Miami, Miami, 33136 USA; 8 National Center for Microscopy and Imaging Research (NCMIR), University of California San Diego, San Diego, 92093 USA; 9 Present Address: Salk Institute for Biological Studies, Molecular and Cell Biology Laboratory, San Diego, 92037 USA

**Keywords:** Physiology, Pre-diabetes, Cellular imaging

Correction to: *Nature Communications* 10.1038/s41467-019-11517-x, published online 28 August 2019.

The original version of this Article contained an error in the author affiliations.

Per-Olof Berggren was incorrectly associated with Universidade Federal do Rio de Janeiro (UFRJ) and CNPq, Rio de Janeiro, Brazil, rather than Miller School of Medicine, University of Miami, Miami, USA

This has now been corrected in both the PDF and HTML versions of the Article.

