# Supplementary material for: Structural basis for delta cell paracrine regulation in pancreatic islets
Source: Nat Commun. 2019 Aug 16;10:3700. doi: 10.1038/s41467-019-11517-x (PMC6697679; doi:10.1038/s41467-019-11517-x)
Supplement: Supplementary file 1 — Supplementary Information [file 41467_2019_11517_MOESM1_ESM.pdf]

Supplementary Information

Structural basis for delta cell paracrine regulation in pancreatic islets

Arrojo e Drigo et al.

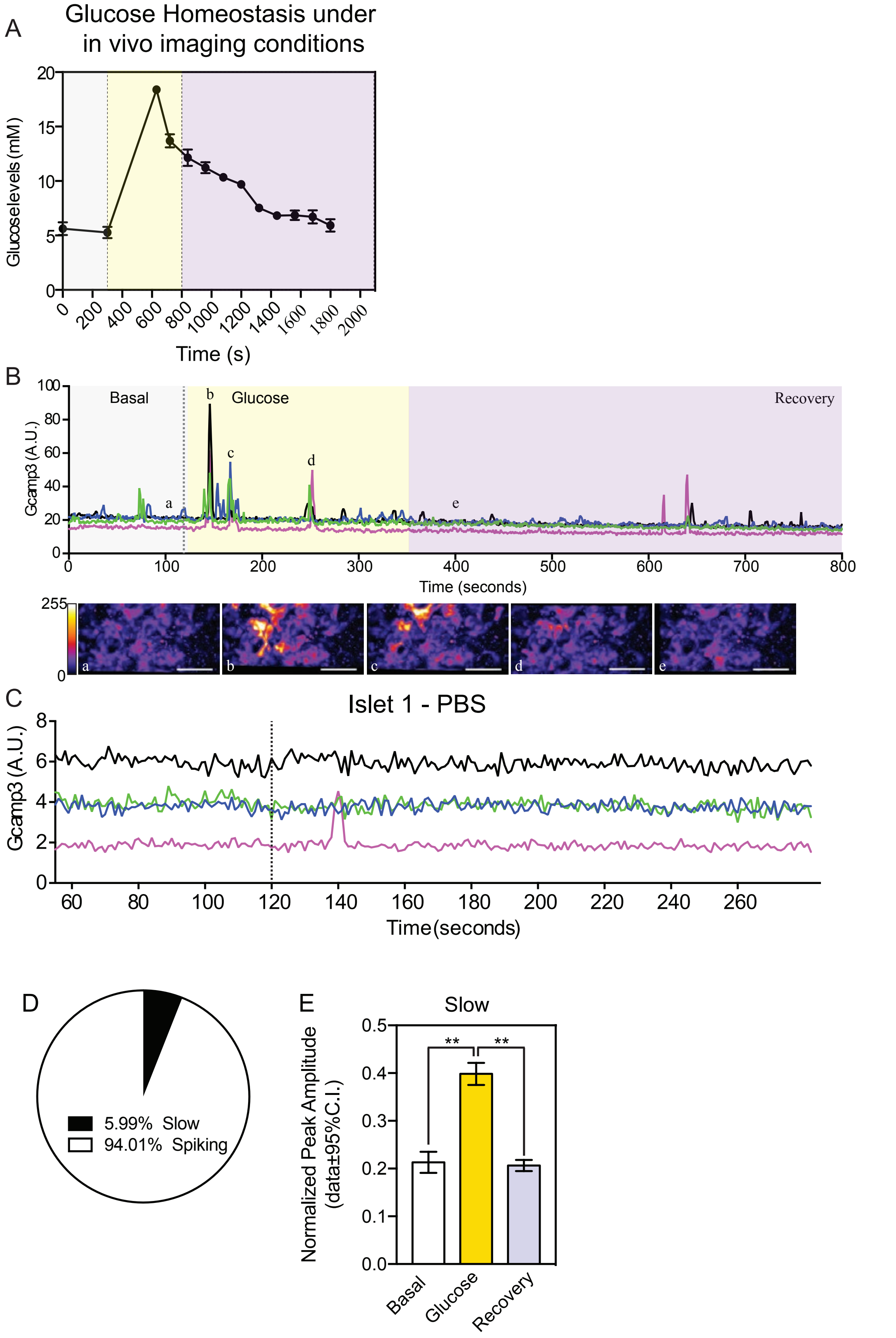

Supplementary Figure 1 – Individual delta cell activity in vivo (A) Glucose homeostasis in mice under in vivo anesthesia conditions used for SST-GCaMP3 imaging (details methods). Basal, glucose and recovery time points (described in Figure 1) are highlighted by grey, yellow and purple shades, respectively. (B) Individual traces from four representative delta cell regions within a single islet in vivo. Glucose (0.4g/kg) was injected i.v. 120 seconds after start of imaging. Grey, yellow or purple shades indicate pre-determined time zones used for data analysis and mark basal, glucose and recovery time periods, respectively. Small cap letters (a-e) shown in (B) represent different delta cell activity events shown on the images arranged below the activity graph. (C) Individual traces from four representative delta cell regions within a single islet in vivo from a mouse injected i.v. 120 seconds after start of imaging with PBS. (D) Pie chart graph showing the relative percentage of spiking (n=217) and slow (n=13) delta cells imaged in vivo. (E) Normalized spiking delta cell activity peak amplitude for basal (n=175 peaks); glucose (n=457 peaks) and recovery (n=477 peaks) time periods. Normalized spiking delta cell activity peak amplitude for basal (n=4574 peaks); glucose (n=7850 peaks) and recovery (n=10518 peaks) time periods. Statistics shown for false discovery rate (FDR, q<0.005 for discovery): **q<0.0001, p<0.0001 by OneWay Anova with a multi-comparison test using a two-stage linear step-up procedure of Benjamini, Krieger and Yakutieli. C.I, confidence interval. For (A) and (E), source data are provided as a Source Data file.

Supplementary Figure 2

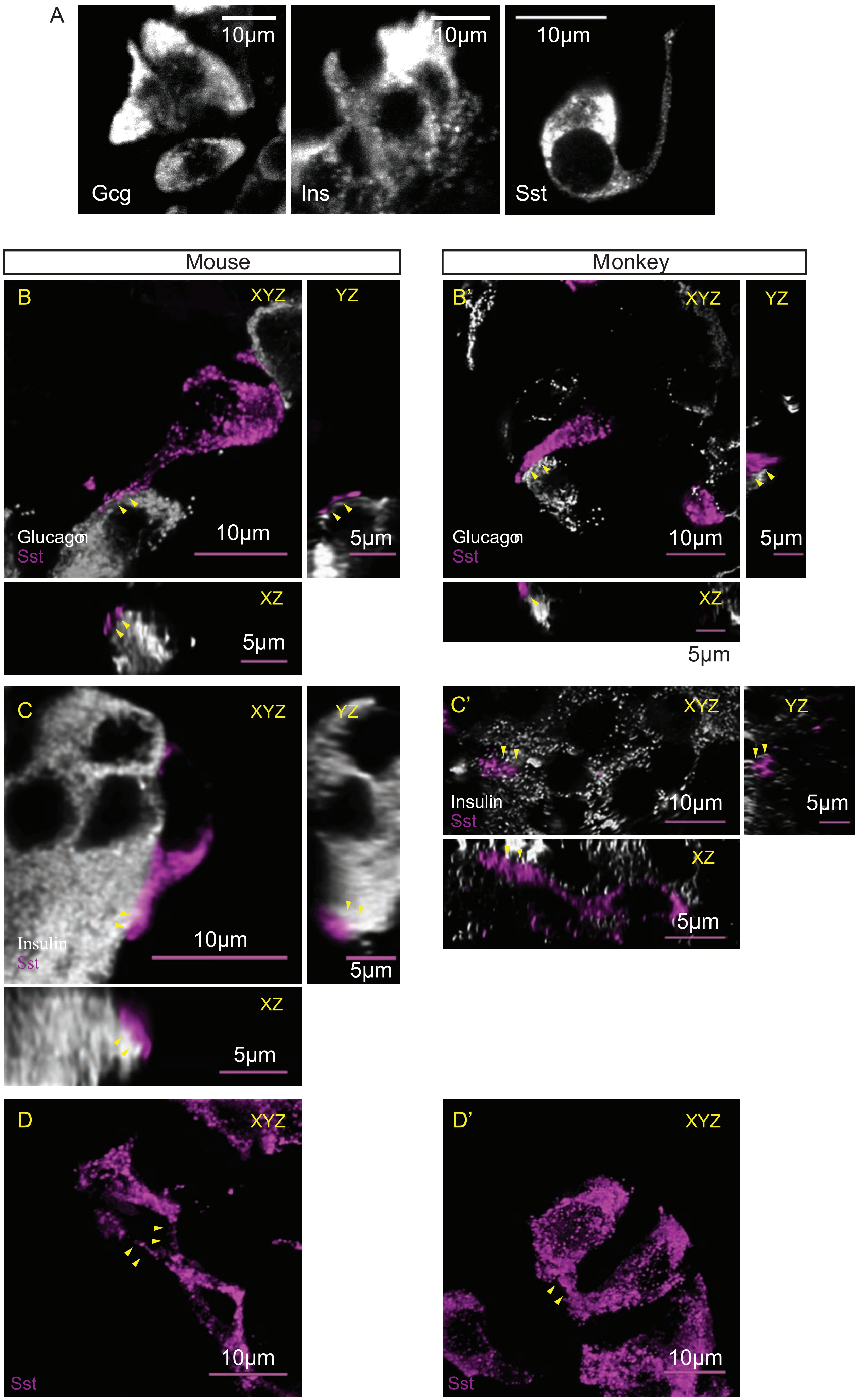

Supplementary Figure 2 – Delta cells have filopodia that reach out to contact neighboring islet cells. (A) Representative maximum projection images of human alpha, beta and delta cells imaged with confocal microscopy of a human islet immunostained with anti-glucagon (Gcg), -insulin (Ins) or -somatostatin (Sst) antibodies. (B-D) Orthogonal views of XY-Z image stacks acquired with confocal microscopy of PFA-fixed mouse and monkey pancreases stained with anti-glucagon (B-B') or anti-insulin (C-C') together with anti-somatostatin (B-D') antibodies. Yellow arrowheads indicate the point of contact between the delta cell filopodia and its target cell. Scale bars: (B-D) XY-Z, 10 µm; XZ and YZ, 5 µm.

Supplementary Figure 3

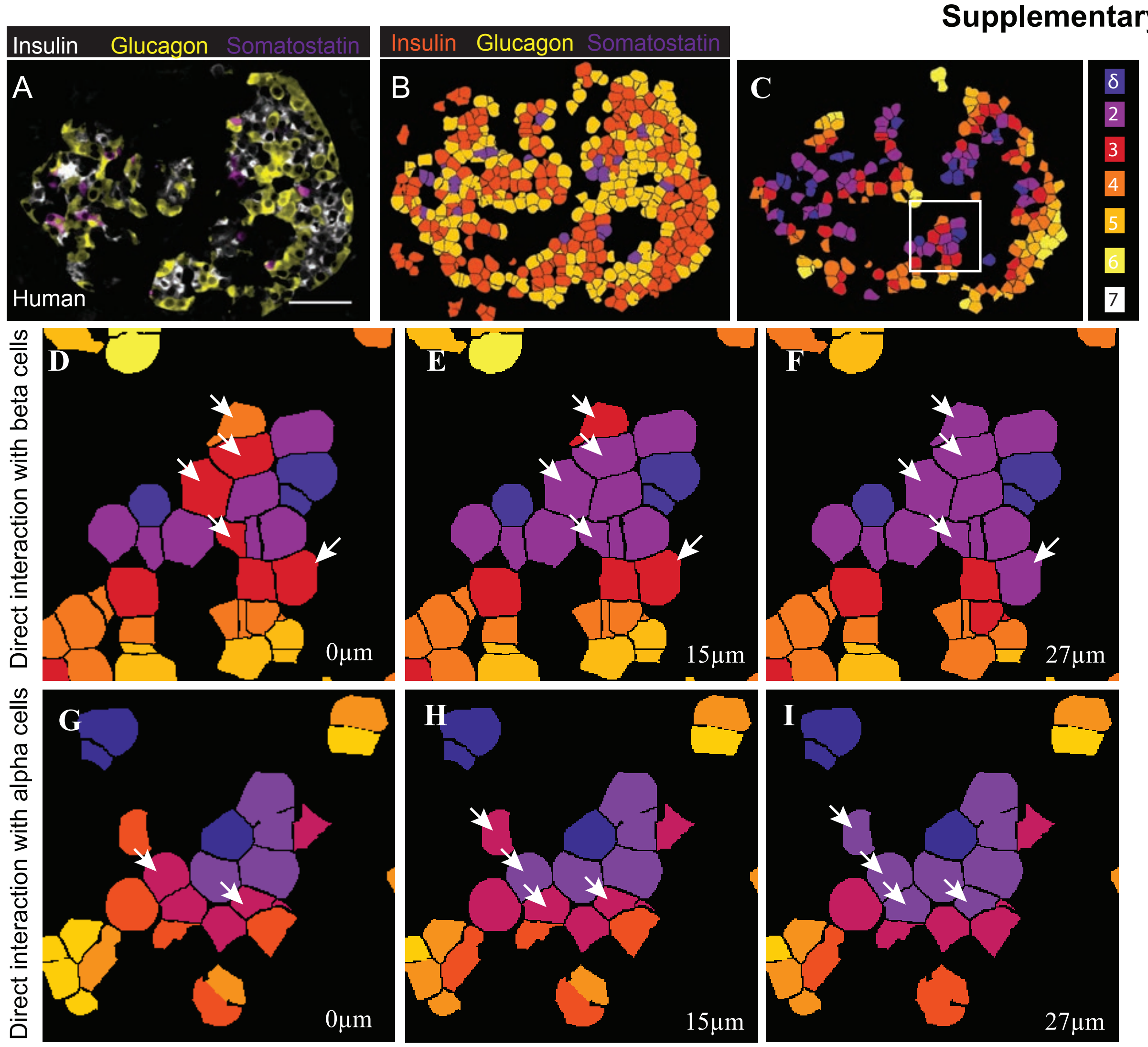

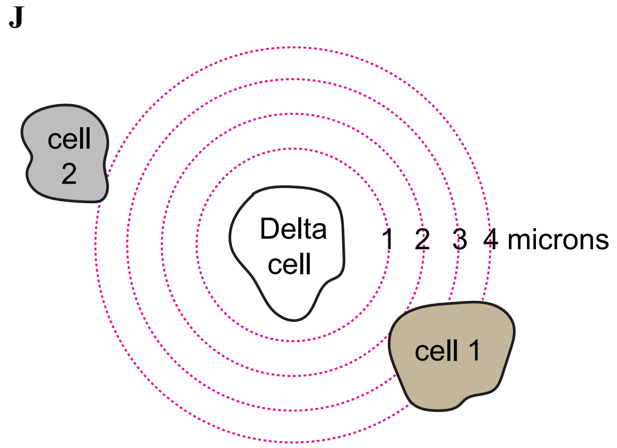

Supplementary Figure 3 – Mapping delta cell contacts in mouse and human islets. (A) Single section image of a human islet immuno-stained with anti-insulin, anti-glucagon and anti–somatostatin antibodies. (B) 3D mapping of beta, alpha and delta cells using the algorithm described in29. (C) Representative distance map of beta cells from the nearest delta cell (purple). (D-I) representative images showing the result of simulation of increasing lengths of delta cell filopodia and its influence on the relative beta cell (D-F) or alpha cell (G-I) contacts in human islets. White arrows indicate cells that are not contacted directly by a target delta cell but become within reach by increasing filopodia length of 15 and 27 μm. (J) Graphical illustration of how the delta cell reach is determined after all alpha, beta and delta cells are mapped. For each delta cell (white, center), we generate radial expansions (pink dotted lines) that increase in diameter with 1 μm steps to simulate the gradual increase in the number of cells that would be within reach of the delta cell filopodia. In this cartoon, cell 1 gets within reach at the 2 μm mark, while cell 2 is reached at the 4 μm mark. In (A), Scale bar, 50μm.

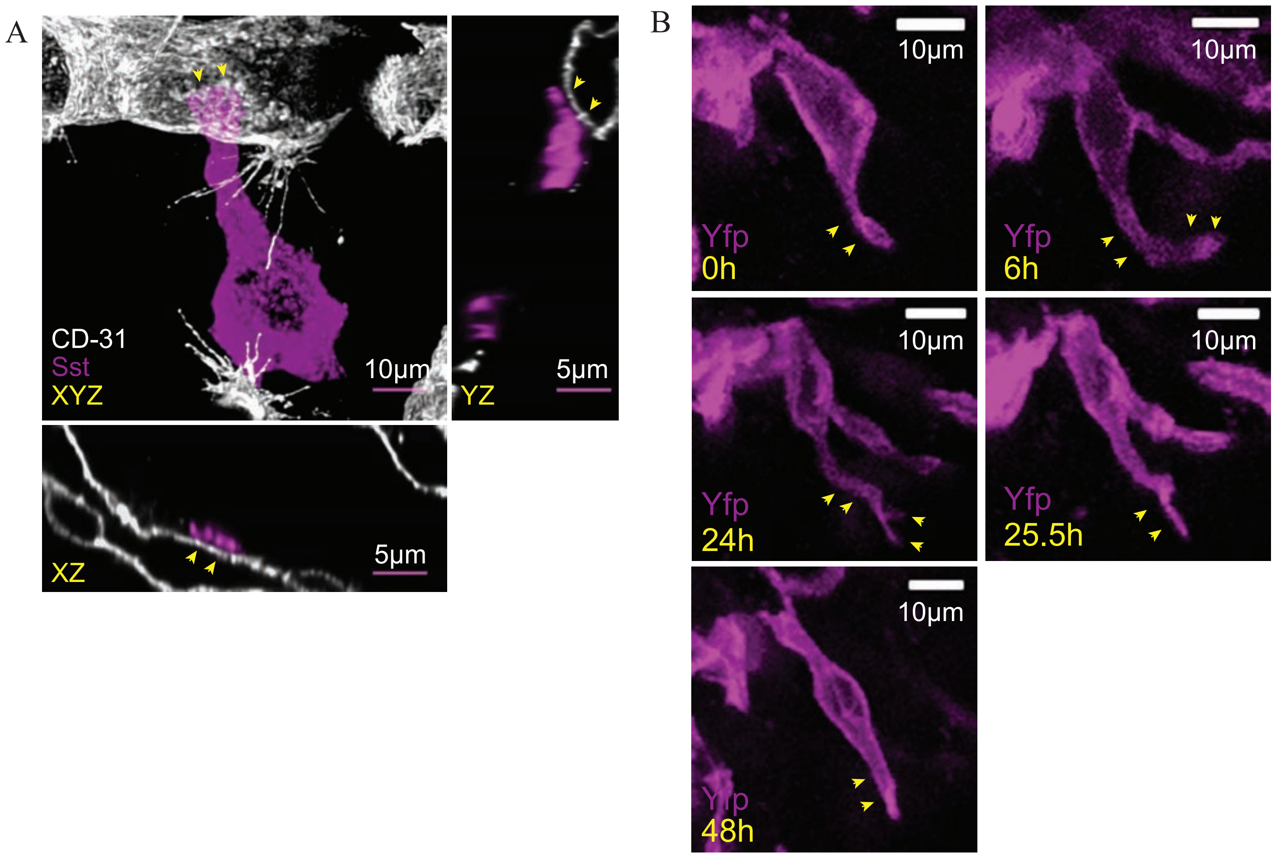

Supplementary Figure 4 – Delta cells can connect to the islet vasculature via their filopodia. The delta cell-vessel connection was validated by immunohistochemistry of PFA-fixed mouse pancreas immuno-stained with anti-CD31 (to mark endothelial cells) and anti–somatostatin antibodies. (A) Orthogonal views of an XY-Z confocal stack with a close-up of a delta cell anchored to a vessel capillary. Yellow arrowheads indicate the point of contact between the delta cell extension and the endothelial cell. (B) 48-hour in vivo follow-up of a single delta cell from an SST-ChR2 islet transplanted into the ACE of a recipient C57/B6 mouse. The time points in which the images were acquired are shown on the left lower corner of each image. Delta cells were visualized by two-photon imaging of YFP. Yellow arrowheads indicate the position of the same delta cell extension during the 48-hour time window. Note the bifurcation of the tip of the delta cell filopopdia at the 24h time point and its absence 1.5 hours later.

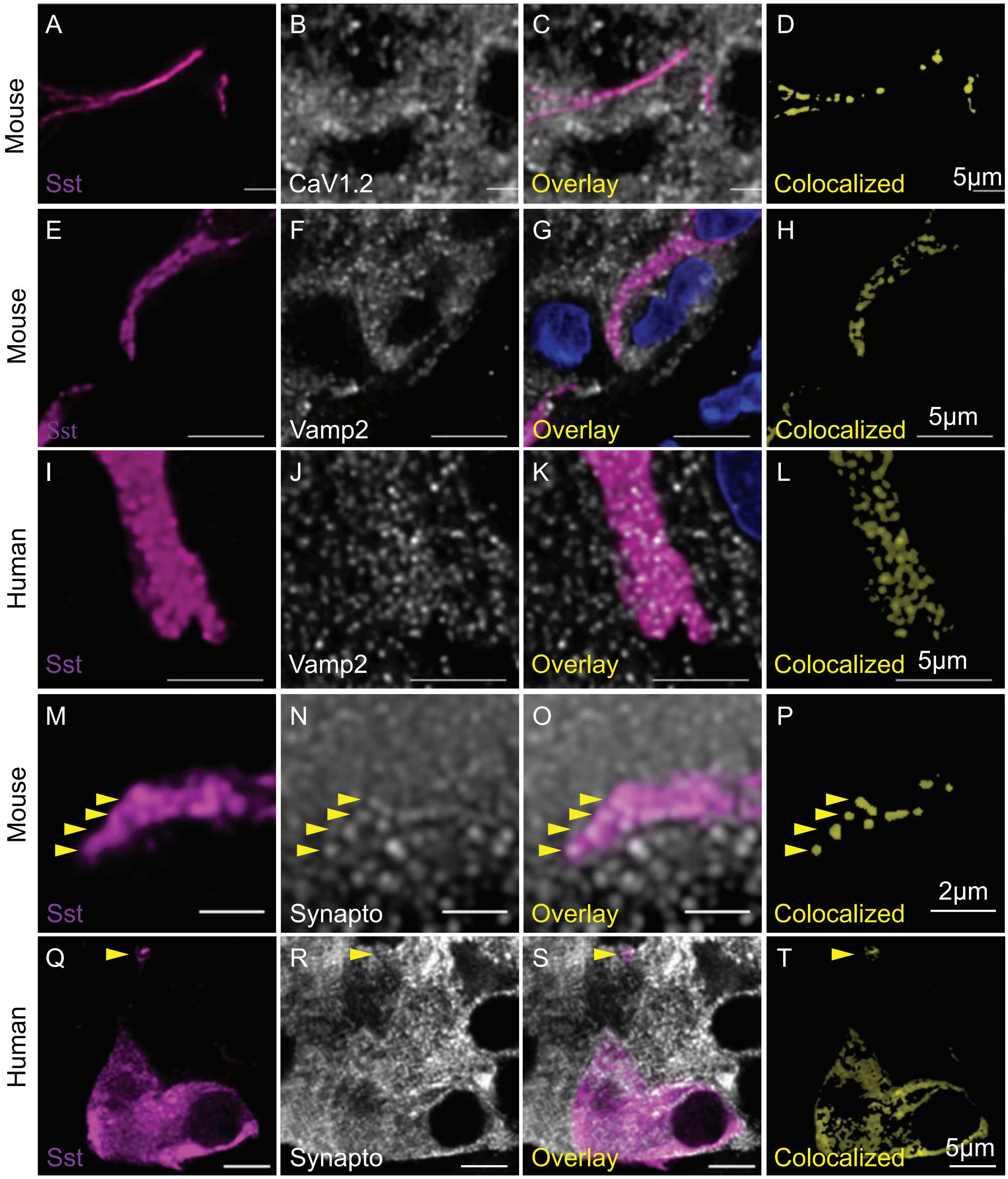

Supplementary Figure 5 – Delta cell filopodia contain somatostatin and elements of an active secretory machinery. (A) Plasma membrane of delta cell visualized by imaging of Yfp, (B) immuno-histochemistry with an anti-CaV1.2 antibody and image overlay are shown in (C). In (D), an image mask showing the overlaying pixels from (A) and (B). (E-F) Close up of a delta cell filopodium from a mouse and (I-K) of a human islet immuno-stained with anti-somatostatin (E and I) and anti-VAMP2 antibodies (F and J). Image overlay is shown in (G and K). (H) and (L), an image mask showing the overlaying pixels from (E-F) and (I-K), respectively. (M-O) Close-up of delta cell filopodia from a mouse and (Q-S) from a human islet immuno-stained with anti-somatostatin (M and Q) and anti-synaptophysin antibodies (N and R). Image overlay is shown in (O and S). (P) and (T), image mask showing the overlaying pixels from (M-N) and (Q-S), respectively. All images are shown as maximum projections and were acquired using Nyquist sampling acquisition parameters and deconvolved prior to co-localization processing (details in online methods section). Scale bars: (A-L) and (Q-T), 5 μm; (M-P), 2 μm.

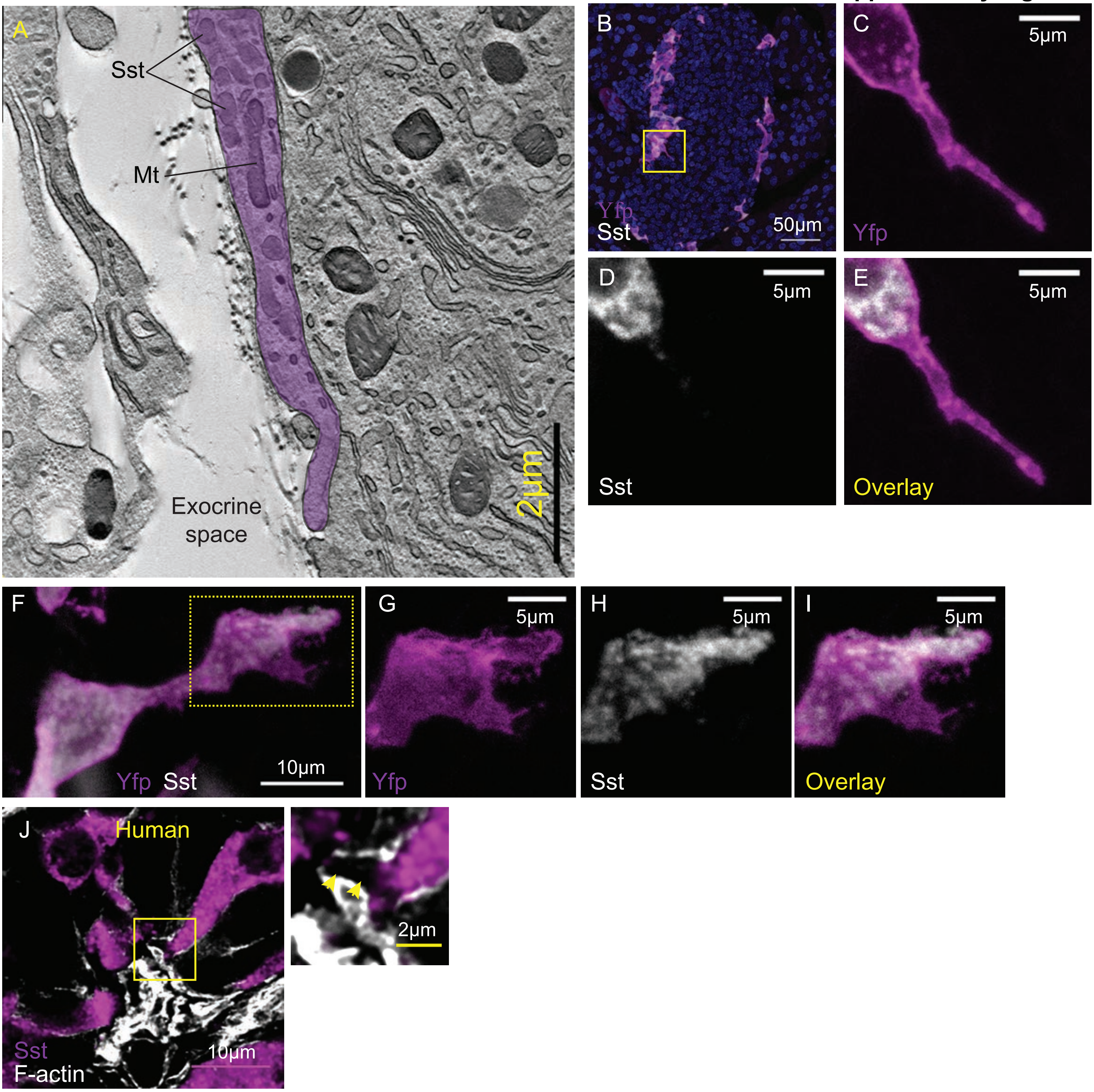

Supplementary Figure 6 – Empty delta cell filopodia in mouse and human islets. (A) Electronic tomogram of a delta cell filopodium in a mouse islet. The filopodium cytoplasm is highlighted by a purple shade. Note the lack of somatostatin towards the end of the filopodium. SST, somatostatin; Mt, mitochondria. (B) Low magnification maximum projection confocal image of a SST-ChR2-YFP mouse islet immuno-stained with anti-somatostatin antibodies (white). The delta cell plasma membrane can be visualized by YFP imaging (pink). (C-D) Close-up images of the area highlighted by the yellow square in (B). Delta cell plasma membrane is shown in (C) and somatostatin content is in (D). (E) Overlay of images shown in (C-D). Note the almost complete absence of somatostatin inside the delta cell filopodium. (F) Similar to (B-E), close-up of a delta cell immuno-stained with anti-somatostatin antibodies (white). Delta cell plasma membrane was visualized with YFP imaging (pink). (G-I) Close-up of a delta cell filopodium highlighted in the yellow square in (F). Note, that while most of the filopodia contain somatostatin, there are filopodia (YFP, (G)) elongating towards the right portion of the image that do not contain somatostatin. (J) Maximum projection confocal image of a human islet immuno-stained with anti-somatostatin antibodies (pink) and F-actin (phalloidin, white). This image shows a delta cell anchored to a vessel (area with high-intensity F-actin signal) through its cellular filopodium. The right inset (J) shows a close-up of the area marked by the yellow square. Yellow arrowheads indicate a large (~1 μm2) area of the delta cell filopodium that does not contain somatostatin. Scale bars: (A), 1 μm; (B), 50 μm; (C-E) and (G-I), 5 μm; (F), 10 μm; (J) 10 μm; inset of (J), 2 μm.

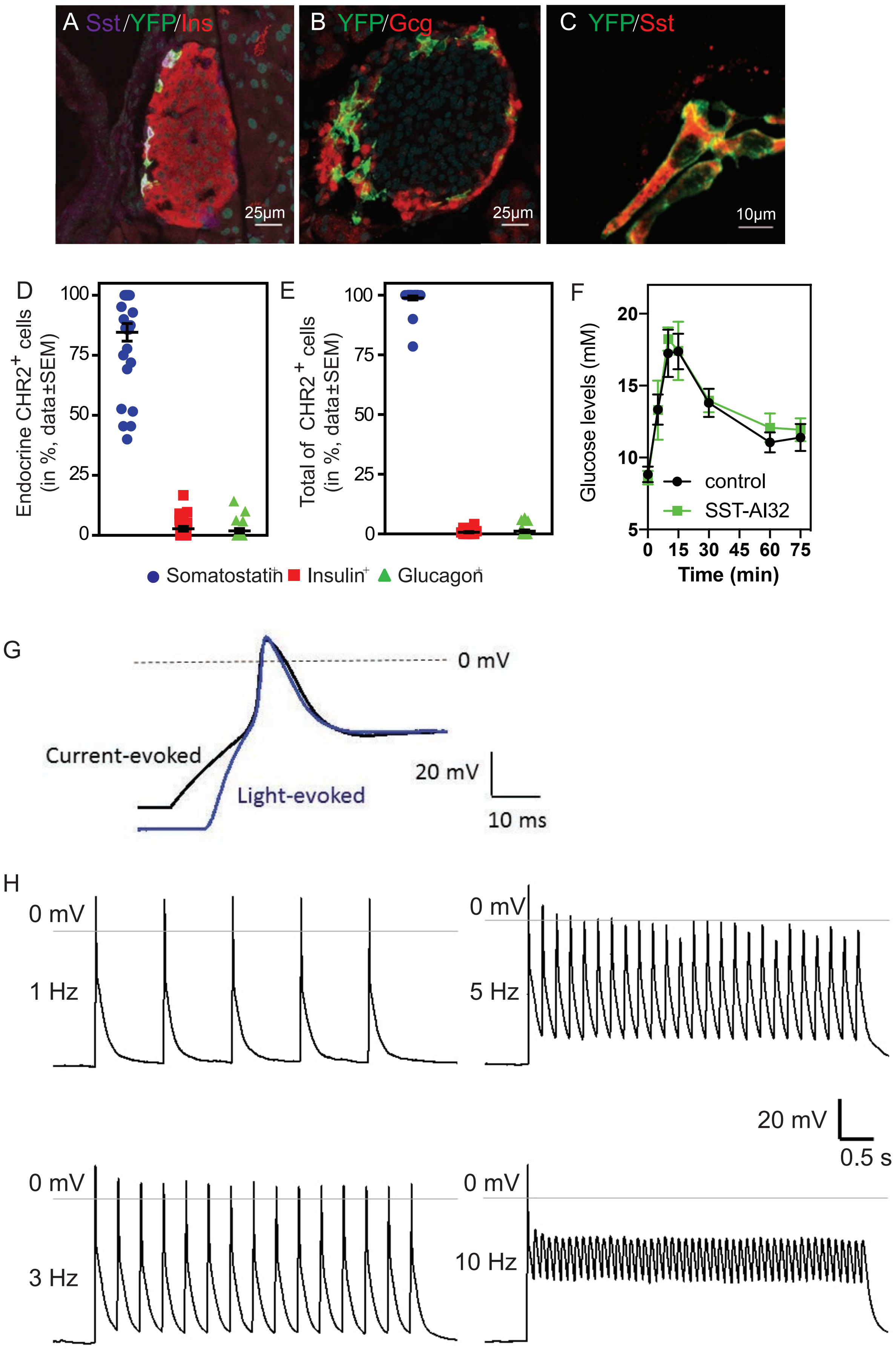

Supplementary Figure 7 – SST-ChR2 mouse glucose homeostasis, pancreas Immunohistochemistry and delta cell ChR2 optogenetics. (A), a representative SST-ChR2 islet immunostained with anti-somatostatin and insulin antibodies and in (B), with anti-glucagon antibodies. In (C), a close up of an SST-ChR2 mouse delta cell stained with anti-somatostatin antibodies. The delta cell plasma membrane can be visualized by imaging YFP. (D) Relative number of delta, beta and alpha cells that are positive for ChR2 in islets from SST-ChR2 mice. (E) Identity of ChR2-positive cells. (F) Glucose tolerance test (GTT) of male and female SST-ChR2 mice (n=3) versus control Sst-CRE or ChR2 (Ai32) littermates (n=10). Scale bar (A-B), 25 µm and (C), 10 µm). (G) Comparison of action potentials evoked by light (blue) and depolarizing current (black) evoked in the same delta cell. (H) Action potentials in ChR2-expressing delta cells induced by light flashes (10 ms duration) applied at different frequencies. Action potentials were reliably evoked at frequencies up to 5 Hz. Dashed lines in (G) and (H) indicate 0 mV. For (F), source data are provided as a Source Data file.

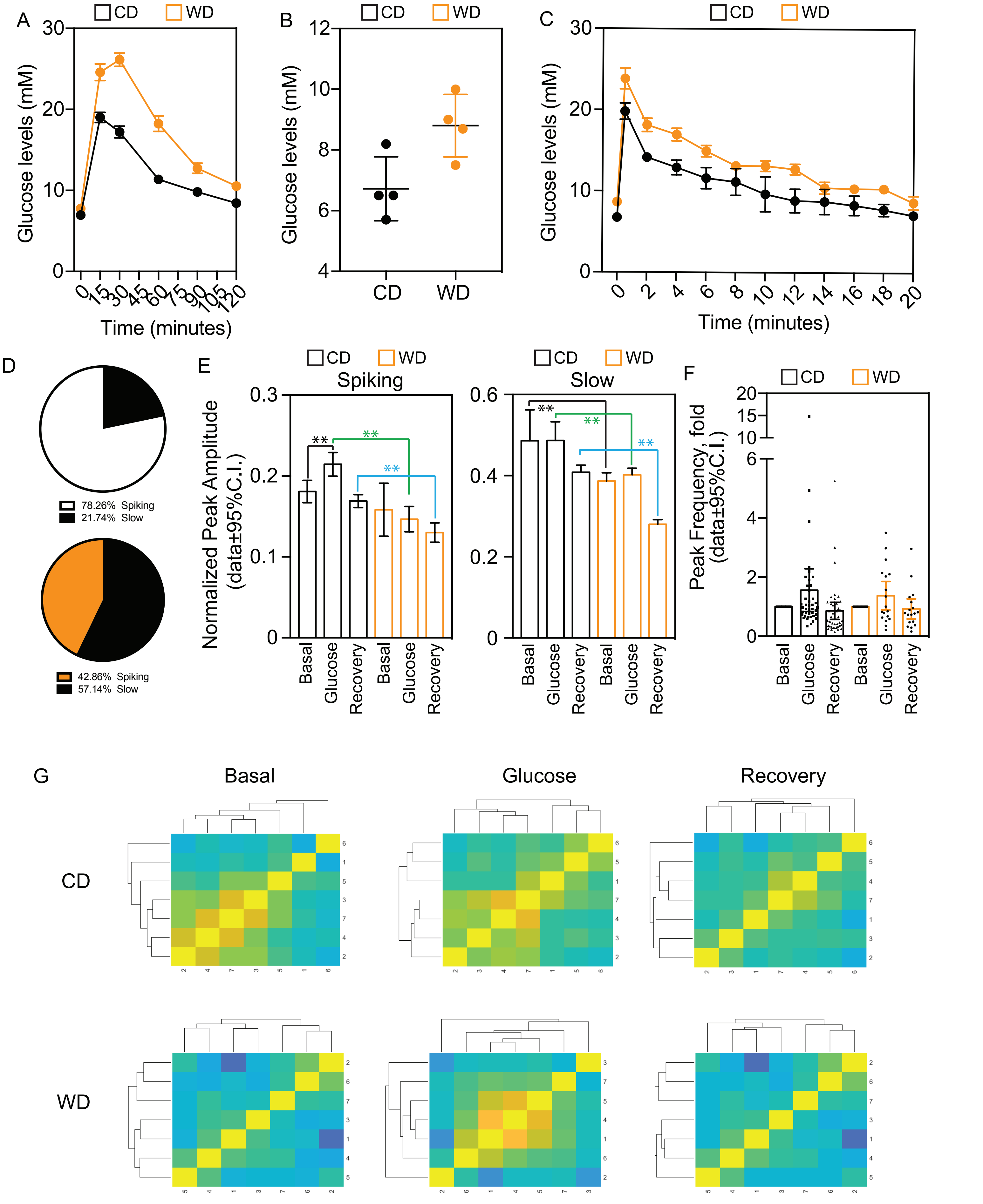

Supplementary Figure 8 – Glucose homeostasis and in vivo delta cell activity in CD- or HFD-fed mice. (A) Glucose homeostasis (IPGTT) and in (B), resting glucose levels of CD (black lines/dots) and HFD-fed animals (orange lines/dots). (C) Glucose homeostasis (IVGTT) under the anesthesia protocol used for in vivo SST-GCaMP3 imaging in CD (black lines) and HFD (orange lines) animals. (D) Pie chart graph showing the relative percentage of spiking (CD, n=18; HFD, n=6 cells) and slow delta cells (CD, n=5; HFD: n=8 cells) imaged in vivo. (E) Normalized [Ca2+]i peak amplitude and (F) peak frequency of spiking and oscillatory (or slow) delta cells under basal, glucose or recovery time periods in CD and HFD-fed animals. (G) Representative hierarchical cluster analysis (HCA) of delta cell activity in CD and HFD islets at basal, glucose or recovery time periods. In (E) data shown is controlled for false discovery rate (FDR, $q<0.006$ for discovery): **$q<0.001$, $p<0.001$ by OneWay Anova with a multi-comparison test using a two-stage linear step-up procedure of Benjamini, Krieger and Yakutieli. C.I, confidence interval. For (A-C) and (F), source data are provided as a Source Data file.

Supplementary Figure 9

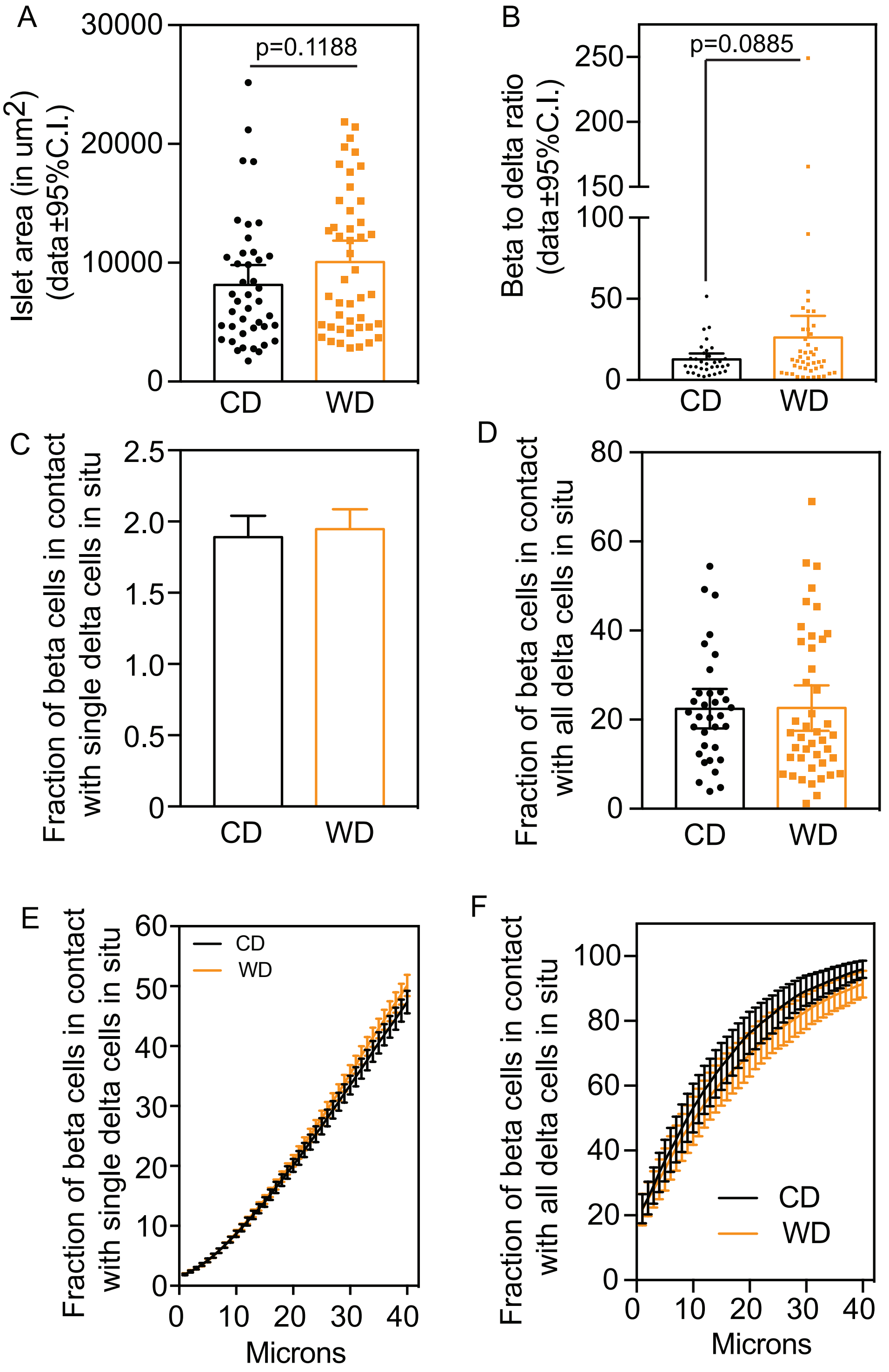

Supplementary Figure 9 – Islet and delta cell filopodia metrics in CD and HFD-fed animals. (A) Islet area in situ from CD- (n=42 islets, n=4 mice) and HFD (n=45 islets, n=4 mice)-fed mouse pancreas. Islet area was calculated by tracing the perimeter of each islet immuno-stained with anti-insulin and anti-somatostatin antibodies and in maximum projection images. (B) Beta to delta cell ratio calculated after automatic identification of beta and delta cells in confocal stacks of CD (n=33 islets, 4 animals) or HFD (n=45 islets, 4 animals) islets. (C) Total fraction (in percentage) of beta cells directly contacted by a single average delta cell. (D) Simulation of delta cell filopodia elongation (from 1 to 40um) and the total fraction (in percentage) of beta cells directly contacted by single delta cells. (E) Total fraction (in percentage) of beta cells directly contacted by all delta cells in a given islet. (F) Simulation of delta cell filopodia elongation (from 1 to 40um, data from Fig 4) and the total fraction (in percentage) of beta cells within the outreach of all delta cells in a given islet in CD- (n=618 delta cells) and HFD-fed (n=918 delta cells) mice. In (A-F), all data are shown as +/- 95% of the confidence interval (C.I.). For (A-F), source data are provided as a Source Data file
