# Supplementary material for: Structural basis for delta cell paracrine regulation in pancreatic islets
Source: Nat Commun. 2019 Aug 16;10:3700. doi: 10.1038/s41467-019-11517-x (PMC6697679; doi:10.1038/s41467-019-11517-x)
Supplement: Supplementary file 3 — Description of Additional Supplementary Files [file 41467_2019_11517_MOESM3_ESM.pdf]

Description of Additional Supplementary Files

File name: Supplementary Data 1

Description: One-way ANOVA multi-comparison and the false discovery rate (FDR) test from the evaluation of the effect of IGF1, VEGF and insulin signaling pathways on the length of the delta cell filopodia in isolated islets.

File name: Supplementary Data 2

Description: One-way ANOVA multi-comparison with Tukey test from the evaluation of the effect of IGF1, VEGF and insulin signaling pathways on the length of the delta cell filopodia in isolated islets.

File name: Supplementary Movie 1

Description: In vivo imaging of GCaMP3 in delta cells in animals under resting conditions. Raw imaging data was acquired at a rate of 1 frame (covering 1 islet volume) per second (fps). For display purposes, the data in this movie is shown at 15 fps.

File name: Supplementary Movie 2

Description: In vivo imaging of GCaMP3 in delta cells before and after glucose stimulation. This movie is representative of the data shown on Figure 1. Raw imaging data was acquired at a rate of 1 frame (covering 1 islet volume) per second (fps). For display purposes, the data in this movie is shown at 20 fps. The labels on the top left represent different time points used for analysis: "basal", before glucose injection; "glucose", i.v. injection of glucose; and "3", representing the recovery period.

File name: Supplementary Movie 3

Description: 3D projection of delta cell from a Sst-ChR2 mouse. This movie is representative of the data described on Figure 2 and shows the 3D rendering of a delta cell from a pancreas from a Sst-ChR2 mouse immune stained with anti-Sst antibodies. Confocal microscopy imaging was used to image ChR2-YFP and visualize the delta cell plasma membrane (shown in white). The delta cell Sst content is shown in green.

File name: Supplementary Movie 4

Description: 3D projection of two delta cells in contact with the islet vasculature. This movie is representative of the data described on Figure 2 and Extended Data 4. This movie shows the 3D rendering from two delta cells in a mouse islet imaged with confocal microscopy of a pancreatic section immunostained with anti-CD31 and Sst antibodies to stain vessels and delta cells, respectively. Vessels are shown in red and delta cells are shown in green.
