# Supplementary material for: Structural basis for delta cell paracrine regulation in pancreatic islets
Source: Nat Commun. 2019 Aug 16;10:3700. doi: 10.1038/s41467-019-11517-x (PMC6697679; doi:10.1038/s41467-019-11517-x)
Supplement: Supplementary file 10 — Reporting Summary [file 41467_2019_11517_MOESM10_ESM.pdf]

nature research

Corresponding author(s): Per-Olof Berggren

Last updated by author(s): Jun 17, 2019

# Reporting Summary

Nature Research wishes to improve the reproducibility of the work that we publish. This form provides structure for consistency and transparency in reporting. For further information on Nature Research policies, see Authors & Referees and the Editorial Policy Checklist.

## Statistics

For all statistical analyses, confirm that the following items are present in the figure legend, table legend, main text, or Methods section.

| n/a | Confirmed | |
|---|---|---|
| ☐ | ☒ | The exact sample size ($n$) for each experimental group/condition, given as a discrete number and unit of measurement |
| ☒ | ☐ | A statement on whether measurements were taken from distinct samples or whether the same sample was measured repeatedly |
| ☐ | ☒ | The statistical test(s) used AND whether they are one- or two-sided<br>*Only common tests should be described solely by name; describe more complex techniques in the Methods section.* |
| ☒ | ☐ | A description of all covariates tested |
| ☐ | ☒ | A description of any assumptions or corrections, such as tests of normality and adjustment for multiple comparisons |
| ☐ | ☒ | A full description of the statistical parameters including central tendency (e.g. means) or other basic estimates (e.g. regression coefficient) AND variation (e.g. standard deviation) or associated estimates of uncertainty (e.g. confidence intervals) |
| ☐ | ☒ | For null hypothesis testing, the test statistic (e.g. $F$, $t$, $r$) with confidence intervals, effect sizes, degrees of freedom and $P$ value noted<br>*Give P values as exact values whenever suitable.* |
| ☒ | ☐ | For Bayesian analysis, information on the choice of priors and Markov chain Monte Carlo settings |
| ☒ | ☐ | For hierarchical and complex designs, identification of the appropriate level for tests and full reporting of outcomes |
| ☒ | ☐ | Estimates of effect sizes (e.g. Cohen's $d$, Pearson's $r$), indicating how they were calculated |

*Our web collection on statistics for biologists contains articles on many of the points above.*

## Software and code

Policy information about availability of computer code

Data collection: *Provide a description of all commercial, open source and custom code used to collect the data in this study, specifying the version used OR state that no software was used.*

Data analysis: *Provide a description of all commercial, open source and custom code used to analyse the data in this study, specifying the version used OR state that no software was used.*

For manuscripts utilizing custom algorithms or software that are central to the research but not yet described in published literature, software must be made available to editors/reviewers. We strongly encourage code deposition in a community repository (e.g. GitHub). See the Nature Research guidelines for submitting code & software for further information.

## Data

Policy information about availability of data

All manuscripts must include a data availability statement. This statement should provide the following information, where applicable:
- Accession codes, unique identifiers, or web links for publicly available datasets
- A list of figures that have associated raw data
- A description of any restrictions on data availability

*Provide your data availability statement here.*

# Field-specific reporting

Please select the one below that is the best fit for your research. If you are not sure, read the appropriate sections before making your selection.

☒ Life sciences ☐ Behavioural & social sciences ☐ Ecological, evolutionary & environmental sciences

# Life sciences study design

All studies must disclose on these points even when the disclosure is negative.

| | |
|---|---|
| Sample size | No statistical method was used to pre-determine the n of animals used for in vivo recordings. In turn, we used the n of animals (or islets) commonly used (n=5-21) in in vivo experiments published by us and others (Refs20-22) |
| Data exclusions | No data was excluded |
| Replication | Each experiment was reliably repeated across different animals/islets from mice and humans. Heterogeneous cellular responses (when found) are shown in the results section (Figs 1-4) and discussed in the body of the manuscript. |
| Randomization | For measurements of the delta cell filopodia, fixed mouse or human islet samples were analyzed using an automated segmentation tool described previosly (Ref.29) thus preventing cell counting bias. All raw in vivo calcium traces were analyzed using previously published (i.e. CalmAn, Ref.48)) and custom scripts (this paper). Images were analyzed using the same parameters for peak detection (datials in methods section). |
| Blinding | Investigators were blinded for the analysis of experiments shown in Figure 2H. |

# Reporting for specific materials, systems and methods

We require information from authors about some types of materials, experimental systems and methods used in many studies. Here, indicate whether each material, system or method listed is relevant to your study. If you are not sure if a list item applies to your research, read the appropriate section before selecting a response.

## Materials & experimental systems

| n/a | Involved in the study | |
|---|---|---|
| ☐ | ☒ | Antibodies |
| ☒ | ☐ | Eukaryotic cell lines |
| ☒ | ☐ | Palaeontology |
| ☐ | ☒ | Animals and other organisms |
| ☒ | ☐ | Human research participants |
| ☒ | ☐ | Clinical data |

## Methods

| n/a | Involved in the study | |
|---|---|---|
| ☒ | ☐ | ChIP-seq |
| ☒ | ☐ | Flow cytometry |
| ☒ | ☐ | MRI-based neuroimaging |

## Antibodies

| | |
|---|---|
| Antibodies used | The following primary antibodies were used: guinea pig anti-insulin (pancreas 1:400, isolated islets 1:1500, Dako - A0564), mouse anti-glucagon (1:400, Sigma Aldrich – G2654), rat anti-somatostatin (pancreas, 1:400 Millipore – AB5494, isolated islets 1:700, BioRad 8330-0009), goat anti-CD31 (1:100, RD Systems – AF3628), rabbit anti-VAMP2 (1:100, Cell Signaling - 13508S), rabbit anti-synaptophysin (1:100, Abcam - ab32594) and rabbit anti-CaV1.2 (1:50, Alomone – ACC-003). Secondary antibodies were raised in donkey and were conjugated to Alexa 488, 546, 561 or 647 fluorophores. |
| Validation | All antibodies used here have been validated previously:<br>Dako - A0564 (discontinued) - Anti-insulin. The following information in from Jackson laboratories - MGI project: This antibody is an un-fractionated guinea pig antiserum. This antibody cross-reacts with insulin from several mammalian species, although it has been optimized for use on human tissues (References listed in http://www.informatics.jax.org/antibody/key/1494).<br><br>Sigma G2654 - Anti-glucagon. Reacts with pancreatic glucagon in RIA and immunocytochemistry. The affinity constant of 6.1 x 10(8) L/M in RIA. The antibody weakly cross-reacts with gut glucagon (enteroglucagon) in an immunohistological assay. Cross-reactivity has been observed with glucagon-containing cells in fixed sections of pancreas from human, porcine, dog, rabbit, mouse, rat, guinea pig, and cat. More details here: https://www.sigmaaldrich.com/catalog/product/sigma/g2654?lang=en&region=US<br><br>Millipore - AB5494 and BioRad 8330-0009 - anti-somatostatin clone YC7. Affinity purified polyclonal antibody in buffer containing PBS with 1% BSA and 0.1% sodium azide. Application notes and validation are found here: http://www.emdmillipore.com/US/en/product/Anti-Somatostatin-Antibody,MM_NF-AB5494<br><br>RD systems - AF3628: Polyclonal goat antibody. Detects mouse CD31/PECAM-1 in direct ELISAs and Western blots. In direct ELISAs and Western blots, approximately 10% cross-reactivity with recombinant human CD31 and recombinant porcine CD31 is observed. Detects mouse CD31 and rat CD31 in flow cytometry. Application notes and validation here: https://www.rndsystems.com/products/mouse-rat-cd31-pecam-1-antibody_af3628<br><br>Cell Signaling - 13508S: VAMP2 (D6O1A) Rabbit mAb recognizes endogenous levels of total VAMP2 protein. This antibody does not cross-react with VAMP1 protein and recognizes VAMP2 protein by IF-IC analysis in mouse and rat cells. More details here: https://www.cellsignal.com/products/primary-antibodies/vamp2-d6o1a-rabbit-mab/13508 |

Abcam - ab32594: Rabbit polyclonal to Synaptophysin. Made using the following synthetic peptide sequence: GPGGYGPQDSYGPQGGYQPD, which corresponds to amino acids 253-272 of Rat Synaptophysin More details and validation here: https://www.abcam.com/synaptophysin-antibody-ab32594.html

Alomone - ACC-003: Rabbit polyclonal antibody directed against an epitope of the rat CaV1.2 protein. The antibody can be used in western blot, immunoprecipitation, immunohistochemistry, immunocytochemistry, and indirect flow cytometry applications. It has been designed to recognize CaV1.2 from mouse, rat, and human samples. More details and validation here: https://www.alomone.com/p/anti-cav1-2-antibody/ACC-003?cn-reloaded=1

## Animals and other organisms

Policy information about studies involving animals; ARRIVE guidelines recommended for reporting animal research

| | |
|---|---|
| Laboratory animals | Transgenic mouse strains used in this study were from Jackson Laboratories. For in vitro studies, male and female mice were used. For in vivo imaging, female mice were used as donors and recipients. The following strains were used: SST-CRE (Ssttm2.1(CRE)Zjh/J), Ai32-ChR2-YFP (B6;129S-Gt(ROSA)26Sortm32(CAG-COP4*H134R/EYFP)Hze/J) and GCaMP3 (B6;129S-Gt(ROSA)26Sortm38(CAG-GCaMP3)Hze/J). Wild-type (WT) C57/6NTac (In Vivos, Singapore) or C57/BL6J (Charles River, USA) mice were used as hosts of Sst-ChR2 and Sst-GCaMP3 islets, respectively. |
| Wild animals | *Provide details on animals observed in or captured in the field; report species, sex and age where possible. Describe how animals were caught and transported and what happened to captive animals after the study (if killed, explain why and describe method; if released, say where and when) OR state that the study did not involve wild animals.* |
| Field-collected samples | *For laboratory work with field-collected samples, describe all relevant parameters such as housing, maintenance, temperature, photoperiod and end-of-experiment protocol OR state that the study did not involve samples collected from the field.* |
| Ethics oversight | All animal procedures were approved by the Institutional Animal Care and Use Committee (IACUC, protocol number 2013/SHS/816) of the SingHealth system or the Karolinska Instituet (protocol number N34/16). |

Note that full information on the approval of the study protocol must also be provided in the manuscript.
